# Pregnancy and Caries of the Teeth

**Published:** 1881-11

**Authors:** 


### PREGNANCY AND CARIES OF THE TEETH.

During pregnancy many women suffer from caries of the teeth and from dental neuralgia. The calcareous salts required for the development of the fœtal skeleton must be supplied by means of an increased ingestion of these materials on the part of the mother; in default of this augmented consumption, the nutrition of the maternal bony tissues is affected and dental caries is the result. Many pregnant women have a morbid appetite for calcareous and other mineral substances. Preparations of calcium, especially the phosphate and hypophosphite, should, in view of the facts mentioned, be administered to enciente females suffering from dental troubles.—(*Gazz. delle Clin.*) *N. Y. Medical Record.*

